# Estimating the stock size of harbor seals (*Phoca vitulina richardii*) in the inland waters of Washington State using line-transect methods

**DOI:** 10.1371/journal.pone.0241254

**Published:** 2021-06-09

**Authors:** Thomas A. Jefferson, Mari A. Smultea, Eric J. Ward, Barry Berejikian

**Affiliations:** 1 Clymene Enterprises, Lakeside, CA, United States of America; 2 Smultea Environmental Sciences, Preston, WA, United States of America; 3 Conservation Biology Division, Northwest Fisheries Science Center, National Marine Fisheries Service, National Oceanographic and Atmospheric Administration, Seattle, WA, United States of America; 4 Environmental and Fisheries Sciences Division, Northwest Fisheries Science Center, National Marine Fisheries Service, National Oceanographic and Atmospheric Administration, Port Orchard, WA, United States of America; Institute of Deep-sea Science and Engineering, Chinese Academy of Sciences, CHINA

## Abstract

Harbor seals (*Phoca vitulina richardii*) in the inland waters of Washington were reduced by predator control programs in the twentieth century, but stocks have rebounded since being protected in the 1970s. Three management stocks are recognized, but there is little information on their current abundance. We conducted 38,431 km of aerial line-transect surveys throughout the range of these stocks in 2013–2016, sighting a total of 4,678 groups of harbor seals. Line-transect analysis with Beaufort sea state as a covariate provided estimates of the number of seals in the water. We then incorporated tagging data from 15 instrumented seals to develop correction factors, both for seals missed in the water while diving, and those that were on shore. Tagging data were modeled with generalized linear mixed models to provide estimates of the proportions diving and hauled out. After applying these correction factors, we estimated that the Hood Canal stock contained 1,368 seals (CV = 16.8%), the Southern Puget Sound stock contained 1,976 seals (CV = 20.5%), and the Washington Northern Inland Waters stock contained 7,513 seals (CV = 11.5%). This study presents a non-traditional approach to estimating the size of Washington inland waters harbor seal stocks, which may also be applicable to other species for which survey and tagging data are available.

## Introduction

**Fig 1:**
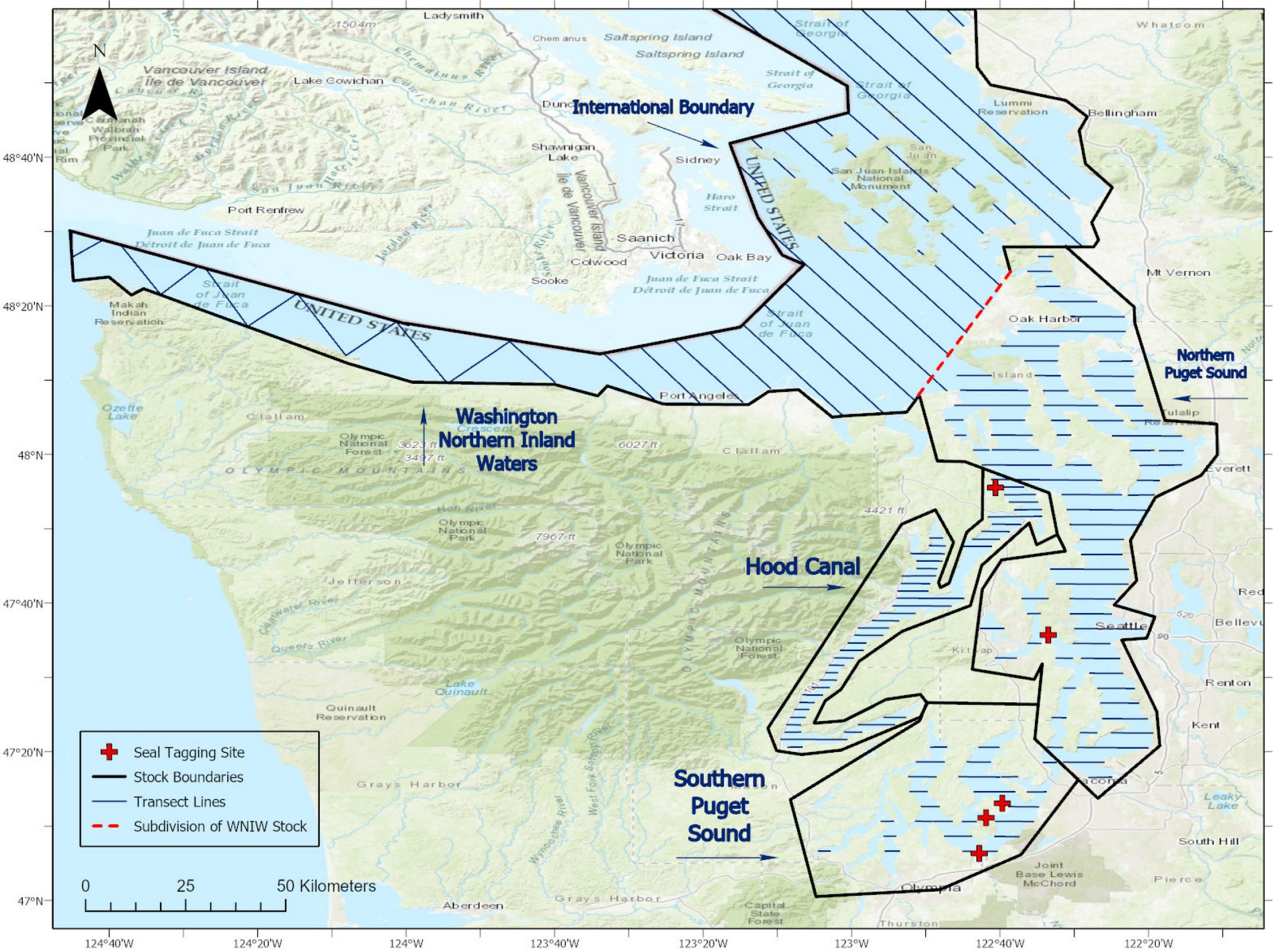
Map of the study area, showing the stock boundaries, planned transect lines, and locations of seal tagging areas. Map was created using ArcGIS® software by Esri. ArcGIS® and ArcMap™ are the intellectual property of Esri and are used herein under license. Copyright © Esri. All rights reserved.

The Pacific harbor seal (*Phoca vitulina richardii*) is the most abundant year-round resident species of marine mammal inhabiting the inland waters of Washington State, including Puget Sound proper, Haro Strait, Strait of Juan de Fuca, and Hood Canal [1, 2]. However, the species was reduced to a small fraction of its original population size by the mid-twentieth century through culling associated with predator control programs to reduce the perceived competition with fishermen for some fish species [3, 4; Fig 1].

Based on genetics, differences in population trends, pupping dates, and spatially variable human impacts, harbor seals in the region are subdivided into three management units (’stocks’) in these waters as recognized by the National Marine Fisheries Service (NMFS) [5]: the Hood Canal, Southern Puget Sound, and Washington Northern Inland Waters stocks (Fig 1). Despite their common and widespread occurrence, population estimates for these seal stocks are quite outdated. The most recent stock estimates were from 1999 based on aerial survey haul-out counts [5]. Though the US Marine Mammal Protection Act (MMPA, 16 USC 1361–1407) dictates that stock estimates for marine mammals should not be more than seven years old [6], a new assessment has not occurred for these three stocks in over 21 years. Up-to-date stock size estimates are needed to evaluate the health of, and trends in, populations to ensure proper management of the species in the face of factors that may adversely affect them. Potential threats to the continued survival of these three stocks in the inland waters of Washington include climate change affecting water conditions and prey, incidental deaths associated with local fisheries, water pollution, and disturbance and displacement associated with increasing human activities and underwater noise, among others [2]. Stock sizes are also needed to estimate the number of individual seals that may be “taken” incidental to US Navy underwater sonar exercises, construction and other activities that may disturb them, as required by the MMPA.

The location of haul-out sites is important for understanding the stock structure and population size for these seals. The Washington Department of Fish and Wildlife (WDFW) has recognized 507 regular haul-out sites used by harbor seals in waters of Washington State [7], with a high degree of haul-out fidelity [8–10]. Haul-out behavior by harbor seals in the region is influenced by age, sex, location, tidal state, time, date, and human disturbance [2, 11, 12].

Pinnipeds have come into conflict with fishermen (both commercial and recreational) in Washington, due to predator/prey interactions with a number of important fish stocks, particularly salmonids. Harbor seals are considered generalist opportunistic predators and appear to adjust their diet to local prey availability [13, 14]. In the inland waters of the Salish Sea, at least 60 different fish species and several crustacean and mollusk species are consumed. Harbor seals eat 14 of the 31 fish species listed with protected status in the Salish Sea [2]. There is growing information and concern that these harbor seals are contributing to the demise of native threatened or endangered salmonid populations in the region [15–17]. Thus, there is a critical need to better understand the spatio-temporal and behavioral overlap of seals and salmonids in terms of feeding preferences of harbor seals and the level of associated impact on listed salmonids. For example, in the 1970s and 1980s, California sea lions (*Zalophus californianus*) learned to prey heavily upon Endangered and Threatened in-migrating salmonids at the Ballard and Chittenden Locks, where the migrating fish were constrained while swimming through artificial fish ladders [18]. Given that these pinnipeds were protected from harm under the MMPA and the salmonids were protected by the US Endangered Species Act (ESA, 16 USC 1531–1544), identification of appropriate management measures was controversial and challenging. Attempts were made by the NMFS and Washington State managers to capture and relocate sea lions, though the animals continued to return to feed annually during the salmonid migrations. The ultimate management action was to kill the sea lions. Continued protection of both salmon under the US Endangered Species act and harbor seals under the US Marine Mammal Protection Act presents similarly complicated management trade-offs that require, at a minimum, accurate estimates of abundance for both predator and prey [19].

Traditionally, population estimates of harbor seals are conducted from fixed-wing aircraft when the highest number of individuals are expected to be hauled out of the water at historical haul-outs along the insular and island coastlines, and on rocks, piers, jetties, sand bars, mudflats, etc. [4]. Each year, the peak of hauling out occurs during the pupping and molting seasons in mid-summer through autumn [e.g., 4, 20]. Counts of harbor seals are then corrected for the proportion of animals estimated to have been missed by survey observers because they were in the water at the time. Haul-out surveys have been conducted periodically in the inland waters of Washington State since 1978 by the WDFW and NMFS [4]. Though not the standard approach used to estimate abundance of most pinnipeds, in-water density and abundance of harbor seals in these waters were estimated based on the number of seals observed in the water during aerial surveys conducted in 2013–2016 on behalf of the US Navy [21, 22]. This line-transect effort was part of a larger survey focused on describing the occurrence, distribution, density, abundance and behavior of all marine mammals, particularly the harbor porpoise *Phocoena phocoena* [22, 23]. These surveys focused on calculating in-water estimates to address the need to enumerate the number of harbor seals that may be exposed to underwater noise associated with US Navy sonar exercises and coastal construction, as required by the MMPA. While corrections were incorporated to address the proportion of in-water animals missed by observers during the surveys, the confidence intervals associated with the abundance estimates were large due to the high degree of variation in dive times reported for the small number of harbor seals tagged at that time.

For this study, our goal was to use fine-scale dive and haul-out data collected concurrently with our 2013–2016 aerial surveys to improve precision associated with trackline detection probability [g(0)]. A related goal was to correct our in-water estimates for seals that were ashore during our surveys, in order to provide a reliable estimate of the entire population size of each of the three harbor seal stocks inhabiting the inland waters of Washington State. To our knowledge, the latter approach has not been previously undertaken. Herein, we present our results, compare our estimates to previous estimates, examine the caveats of our approach, and provide recommendations for future stock estimation.

## Materials and methods

### Study area and period

The study area consisted of the inland waters of Washington State, including Puget Sound proper, Hood Canal, the Strait of Juan de Fuca, Haro Strait, the San Juan Islands area and some nearby waters adjacent to the border of British Columbia (BC), Canada [Fig 1; see also 21, 23]. Four geographic survey strata were identified (1) Northern Waters region (incorporating US waters of the Strait of Juan de Fuca, Haro Strait, and San Juan Islands), (2) Hood Canal, (3) Northern Puget Sound, and (4) Southern Puget Sound (Fig 1). For the purpose of estimating sizes of the three harbor seal management stocks currently recognized by the NMFS (see ***Stock Definition*** below), the Northern Waters region was pooled with the Northern Puget Sound region to match the boundaries of the Washington Northern Inland Waters stock (Fig 1). The Hood Canal and Southern Puget Sound stock boundaries matched those of our other two geographic strata with the same names.

**Fig 2:**
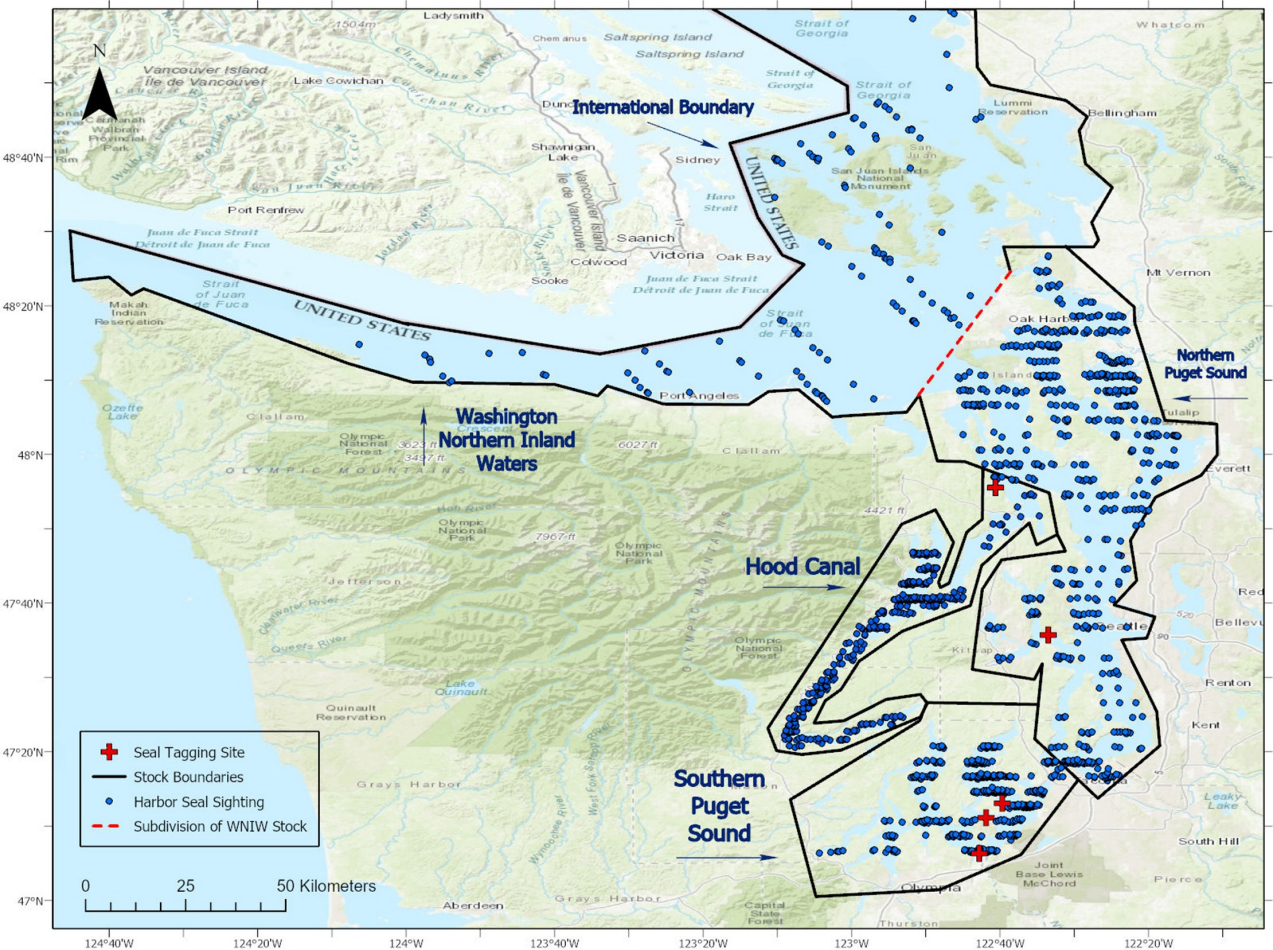
Map showing the locations of harbor seal sightings used for estimating density and abundance in this study (i.e., after filtering). Map was created using ArcGIS® software by Esri. ArcGIS® and ArcMap™ are the intellectual property of Esri and are used herein under license. Copyright © Esri. All rights reserved.

Extensive aerial surveys (38,431 km of observation effort) were conducted over six survey periods (see below) in the Puget Sound and Hood Canal regions from 2013 to 2016 and included all four calendar seasons (Table 1). During April 2015, applying the same field methods, we conducted a 5-day aerial survey (806 km of useable effort) of the Northern Waters region that included adjacent Canadian waters of the southern Strait of Georgia, the San Juan Islands, waters west of Whidbey Island, and the Strait of Juan de Fuca (Fig 1). The addition of this latter period of surveys allowed us to calculate total estimates for the three stocks of interest. Only effort and sightings made within US waters during our surveys are included in this paper, corresponding with the three US harbor seal stock boundaries as identified below (Fig 2).

**Table 1:** Summary of aerial surveys.

Year	Calendar Season	Survey Period	Total Effort (km)
2013	Autumn	30 August-4 September	6360
2014	Summer	21–27 July	5262
2014	Autumn	14–21 September	7678
2015	Winter	5–12 January	618
2015	Spring	15–22 April	8579
2015	Spring	23–27 April	3329
2016	Winter	16–25 January	6605
Total			38,431

### Stock definition

Harbor seals in the inland waters of Washington State have been considered to be distinct from those along the outer coast of Washington, Oregon, and California, based on information on pupping dates [24], individual movements [25, 26], pollutant levels [27], and more recently molecular differences [28, 29]. Within the inland waters of Washington, three management stocks are currently recognized by the NMFS, based largely upon differences in mitochondrial DNA (mtDNA) and microsatellites [28, 29]. These stock boundaries are depicted in Fig 1 and consist of:

1. Southern Puget Sound (south of the Tacoma Narrows Bridge),
2. Hood Canal (south of a line demarcating the entrance to Hood Canal), and
3. Washington Northern Inland Waters (north of the Tacoma Narrows Bridge and the Hood Canal boundary to the Canadian border, and including the Strait of Juan de Fuca).

These three stocks are well established and management of seals occurs accordingly [5]. Although there are certainly movements of seals across the Canadian border, NMFS management is only directed at those seals that occur within US territorial waters. Potential Biological Removals (PBRs; the main management approach for marine mammals used by the NMFS) cannot currently be calculated for these stocks, due to outdated abundance estimates. However, none is thought to be subject to takes above the PBR level, nor is any listed under the ESA, and therefore none of them is considered to be a “strategic” stock by NOAA [5].

### Aerial surveys

Aerial line-transect surveys were conducted for marine mammals from a Partenavia P68-C or a Partenavia Observer high-wing, twin-engine airplane [21, 23]. Pre-determined systematic transect lines running east-west were followed, generally oriented perpendicular to water depth contours. In all areas except Hood Canal, survey lines were spaced 3.7 km apart; however, in 2016, additional lines were added in the Hood Canal region to increase coverage, resulting in 1.8-km line spacing. In the Washington Northern Inland Waters region, survey lines in the San Juan and Gulf Islands closely followed those from a previous 2002–2003 aerial survey for marine mammals [23]; these lines were nonoverlapping, oriented 135° from the vertical, and spaced approximately 5.55 km apart. In the Strait of Juan de Fuca portion of the Northern Waters region, survey lines were spaced about 11.1 km apart and followed an overlapping sawtooth pattern. The different design of the sets of transect lines for the latter two areas (Strait of Juan de Fuca portion of the Northern Waters) had to do with a desire to match transect lines used previously by NMFS in their historical surveys (Fig 1).

One pilot and four professionally trained marine mammal biologists (at least two with over 10 years of related experience) were aboard the aircraft. Two biologists observed from the center seats of the aircraft through bubble windows on each side of the plane. To address line-transect analysis assumptions [30], the third biologist observed directly below the plane through the belly window (located behind the center seat row) to reduce chances that sightings were missed “on or near” the survey line [30]. A data recorder sat in the front right copilot seat. Surveys were flown at a target speed of 185 km/h and altitude of 234 m. When a sighting was perpendicular to the aircraft, a Suunto inclinometer was used to record declination angle readings to sightings. Sighting, effort, and environmental data were entered by the recorder into a laptop computer running Mysticetus™ Observation software (http://mysticetus.com/), which automatically calculated perpendicular distance to the sighting and displayed it on a bathymetric map. Most sightings were recorded in passing mode; only a small number (<10%) of sightings were circled (off-effort) to confirm species identifications.

### Data treatment

Separate Excel™ spreadsheet databases were prepared for the sighting data and effort data. Survey data in each database were filtered with the following criteria used to extract relevant data for the line-transect analyses (as part of an approach to ensure meeting assumptions of line transect theory):

- Only data (e.g., sightings and effort) collected on systematic transect lines (data from transit and connector effort were excluded). “Connector effort” refers to short lines that connect the main transect lines. In most cases, these lines are over land, but even over water, these lines are excluded because the data are often parallel to shore or at a depth contour that leads to issues regarding how representative they are of the density that is being estimated.
- Only data collected in Beaufort sea states (BSS) 0–2 (following the protocol of Ampela et al. [22], which previously analyzed a portion of this dataset).
- Only data without significant glare issues (i.e., “hard” glare within which a marine mammal could not be seen occurring within more than 30% of each of the three observers’ fields of view [0 to 90 degrees left and right of the plane’s nose and the belly window] for more than three minutes]).

Input files for the line transect analyses were then prepared from the filtered data.

### Line transect analysis

We used both conventional line-transect methods (also known as Conventional Distance Sampling or CDS) and multiple-covariate line-transect methods (also known as Multiple Covariate Distance Sampling or MCDS) to analyze the aerial survey data for estimating density and abundance of harbor seals [30–32]. The latter approach is generally preferred, as it uses information on environmental factors that are likely to affect detection probability (such as variables describing sighting conditions), and usually (though not always) produces estimates with higher precision (i.e., lower variances) [32, 33]. However, datasets with small sample sizes (such as often occurs in marine mammal studies) can make it difficult or impossible to achieve model “convergence” in some MCDS analyses; it is thus critical to always start each analysis with CDS methods (this also helps to determine the appropriate truncation distance and overall modeling approach).

Data were analyzed using the software DISTANCE 6.2, Release 1 [34]. Estimates of density and abundance (and their associated coefficient of variation) were calculated using the following standard formulae:

$$\hat{D}=\frac{n\hat{f}(0)\;\hat{E}(s)}{2\;L\hat{g}(0)}$$

$$\hat{N}=\frac{n\hat{f}(0)\;\hat{E}(s)\;A}{2\;L\hat{g}(0)}$$

$$\hat{CV}=\sqrt{\frac{\hat{var}(n)}{n^{2}}+\frac{\hat{var}[\hat{f}(0)]}{{\left[\hat{f}(0)\right]}^{2}}+\frac{\hat{var}[\hat{E}(s)]}{{\left[\hat{E}(s)\right]}^{2}}+\frac{\hat{var}[\hat{g}(0)]}{{\left[\hat{g}(0)\right]}^{2}}}$$

where *D* = density (of individuals),

*n* = number of on-effort sightings,

*f*(0) = probability density function evaluated at zero distance,

*E*(*s*) = expected average group size (using size-bias correction in DISTANCE),

*L* = length of transect lines surveyed on effort,

*g*(0) = probability of detecting seals (in our study, this accounts for both seals missed due to being on a dive, and those that were hauled out at the time of the survey),

*N* = abundance,

*A* = size of the survey area,

*CV* = coefficient of variation, and

*var* = variance.

We produced estimates of density and abundance using the entire filtered dataset, stratified by season and by the four survey regions. Our ultimate objective was to obtain a stock size estimate for each of the three stocks that NMFS recognizes in the MMPA stock assessment reports [5]. We therefore stratified the data to match these stock regions as discussed above. The four solar seasons were defined as follows: summer–June-August, fall—September-November, spring–March-May, and winter–December-February. Final estimates all used the MCDS approach, with Beaufort sea state as a covariate, as this resulted in estimates with the highest level of precision (as determined by the lowest CVs). We used the 02 method for estimating variances, which is recommended for survey designs with systematic transect lines that can accommodate overlapping strata [34].

To avoid potential overestimation of group size, we used the size-bias-adjusted estimate of average group size available in DISTANCE. To facilitate modeling, the Perpendicular Sighting Distance (PSD) data were truncated to remove outliers. We experimented with several different truncation strategies, and settled on the most appropriate one (in terms of both minimizing CVs and examining PSD plots to ensure a good model fit) for the final analyses. We modeled the data with the half-normal (with hermite polynomial and cosine adjustments), and hazard rate (with simple polynomial and cosine adjustments) models. For each survey region, we used a pooled estimate of the probability density function and group size, but we did not pool sighting rates. The model with the lowest value of Akaike’s Information Criterion (AIC) was selected for the final estimates.

We produced two sets of estimates–an uncorrected estimate of only the number of seals in the water, and a second estimate that incorporates a g(0) correction factor for both missed trackline detections (availability portion only) and seals hauled out at the time of the survey (see below under Estimation of Correction Factors). The latter estimate provides an approximation of the total abundance for each stock, which include both seals in the water and those on land. We used seal tagging data to model the correction factors (see below). Presenting both the uncorrected and corrected estimates allows the reader to compare our estimates more easily with those of other line-transect studies, which often do not correct for missed trackline detections.

### Seal tagging

Harbor seal dive and haul-out data were obtained from instrument packs deployed on 15 harbor seals monitored as part of another study investigating the role of harbor seal predation on migrating steelhead smolts in Puget Sound [35]. Seal tagging was done as part of the Salish Sea Marine Survival Project and the data were provided by WDFW under contract. Briefly, the WDFW captured 10 adult males (59 to 118 kg) and 5 adult females (73 to 96 kg) at five haul-outs, including the Nisqually estuary, Gertrude and Eagle Islands in south Puget Sound, Orchard Rocks in Central Puget Sound, and the Colvos Rocks complex in Admiralty Inlet (Fig 1) [35, Supplement 1]. Each seal was fitted with an instrument pack glued to the back of the seal. Deployment dates ranged from 6 April through 5 May 2016 and instruments were configured to immediately begin collecting data. Because the focus of these deployments was to better understand potential predation of steelhead, tags were pre-programmed to transmit every 6 days before April 15 and after July 31, but daily from April 15—July 31. Each instrument pack contained a satellite-linked time depth recorder (TDR) and Fastloc GPS tag (model MK10AF, Wildlife Computers, Redmond, WA, USA, www.wildlifecomputers.com). An acoustic transceiver and a VHF tag were also incorporated into each pack for other purposes [35]. The MK10AF tags stored up to 144 Fastloc GPS locations per day. The instrument packs were recovered from early July 2016 through January 2017, with most being recovered from mid-August through early October 2016. Depth data on the MK10AF tags provided a clear indication of time at which packs became detached from the seals and thus when data no longer reflected seal behavior. The number of seals represented during each month from April through October is indicated in the results. The MK10AF tags were configured to start and end a dive at depths of 1.5 m, but dives less than 3 m or shorter than 20 s were ignored. Haul-out events were determined if the wet/dry sensor was dry for any 30 s within a minute, and a tag was considered in a haul-out state after being dry for 5 consecutive minutes. The tag ‘exited’ a haul-out state if wet for any 45 s within a minute. Data from all 15 seal tags were analyzed as a single group to collectively represent the behavior of harbor seals over a broad area including south, central and northern Puget Sound. More details on the seal tagging work can be found in Moore et al. [35].

### Estimation of correction factors

Trackline detection probability, *g*(0), is assumed to be unity (1.0) in most line–transect studies of pinnipeds, both for aerial [36–40] and shipboard surveys [41–44]. However, ignoring the fact that *g*(0) is in reality less than 1.0 in most aerial surveys can cause serious downward bias, and recently more studies are working to develop estimates of *g*(0) to correct their abundance estimates [22, 45–48]. These studies generally use diving data from tagging studies or double-platform methods.

Trackline detection probability in our study could not be directly estimated from the data collected in the aerial surveys because we did not conduct diving experiments, nor use independent observers. Instead, we used archived tag data from the 15 harbor seal tags to estimate probabilities of animals being hauled out, and probabilities of a seal being in a dive state (dive summaries generated from Wildlife Computers Dive Analysis Version 3.0, Wildlife Computers, Redmond WA). For each seal’s dive data, we calculated the number of minutes per hour that the seal was recorded in a dive state (minimum dive depth = 1.5m), and we did not attempt to categorize the different types of dives [see 49]. We used the wet–dry sensor data as a binary indicator of animals being hauled out at any particular timestamp.

There are a number of complications in joining the haul-out and dive data based on dates and timestamps (haul-out sensors may not log data continuously, resulting in NAs), thus we had to assume independence and model the dive probability separately from the haul-out probability. For both responses, we modeled probabilities using generalized linear mixed models (GLMM) [50]. To avoid potential biases during periods with few seals, we binned the data into weeks (Friday January 1—Thursday January 7 being assigned to week 1, etc.) and removed data during weeks where less than five seals were present. For the haul-out data we used the binary wet/dry data as in a Bernoulli GLMM with a logit link (each timestamp for the wet–dry sensor corresponding to an event), and for the dive data we used a Beta GLMM with a logit link (the fraction of each hour in a dive state as the response). For both models, we used the same fixed and random effects covariates. Following previous work [12], we initially considered using covariates related to tide as predictors of behavior (time to high tide, tidal height, lunar phase). Because of estimated weak effects of these covariates on the probability of individual seals hauling out, we modeled hour of day and week of the year as fixed effect factor variables (shared across animals), and included random effects in individuals (allowing each individual to have a different probability of being hauled out) and random interactions between week and animal (allowing the animal-week to be the grouping factor, so that each animal’s probability of being hauled out varies by week, and in different ways). Estimation was done in a maximum likelihood framework using glmmTMB [4]. Predictions with standard errors were made from each model to all combinations of days and hours in the survey, for an average untagged seal.

To calculate the probability of an animal being ’available’ to the aerial survey, we assumed that:

Pr(available)=[1−Pr(haulout)]∙[1−Pr(dive)]

where *Pr (available)* = probability of being available for detection on the survey,

*Pr (haul-out)* = probability of being hauled out at the time of the survey, and

*Pr (dive)* = probability of being on a dive as aircraft passed overhead.

Using estimates of predictions and standard errors in link-space (scale of the linear predictor) from each model, we generated 95% confidence intervals via Monte Carlo simulation. After drawing 10,000 random values from each quantity, we calculated the mean and CIs on *Pr*(*haulout*), *Pr* (*dive*), and *Pr* (*available*) for each hour and day for which data were available and during which aerial surveys were conducted (predictions made for an average seal without tagging data available, between 0900 and 1700). Correction factors were calculated from the predicted availability estimates for each of the three seasons from which we had tagging data (spring–March-May, summer–June-August, and autumn- September-November). Since tagging data were available only for specific times of the year, we made the assumption that tagging data from a particular season were representative of that season. Due to absence of tagging data, we were unable to calculate a winter correction factor; thus, winter estimates only present the uncorrected in-water estimates.

## Results

### Data collection

The aerial surveys covered a total of 38,431 km of survey effort in US waters of the Salish Sea, and we detected a total of 4,678 harbor seal sightings. After filtering the data (see above), we used 8,040 km of survey effort and 2,437 harbor seal sightings in the line-transect analysis of density and abundance. Truncation of the sighting data left 2,353 sightings remaining to be used in calculating the detection functions.

### Correction factors

**Fig 3:**
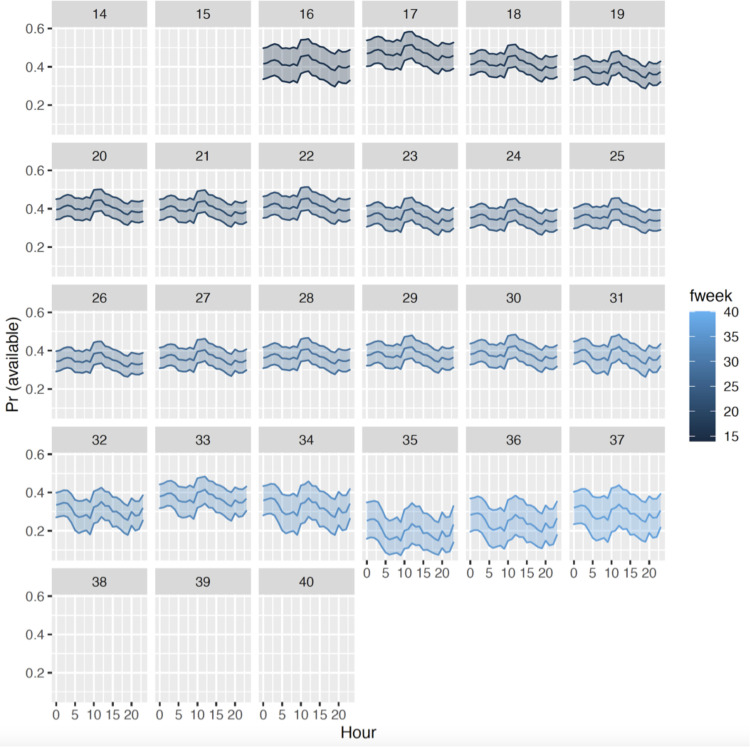
Modeling results of the proportion of seals available to be detected on aerial surveys, shown by week of the year, and time of day. Week numbers 16–21 correspond to spring, 22–34 correspond to summer, and 35–37 correspond to autumn.

Modeled availability probabilities, Pr(*available*), by week and hour of the day is shown in Fig 3. Highest values were generally at mid-day (around noon), and lowest values were in morning and afternoon daylight hours. The availability of seals to being surveyed was highest in the spring months, when seals spend less time hauled out and more time in the water, lowest in the autumn, when seals molt, and intermediate in the summer, when most pupping occurs [4]. The *g*(0) correction factors, which include both the diving component and the hauled-out component, ranged from a low of 0.249 (autumn) to a high of 0.429 (spring; Table 2).

**Table 2:** Correction factors and their associated details by season.

Parameter	Spring	Summer	Autumn
n	54	117	27
Pr(available)	0.43	0.37	0.25
Pr(available) SE	0.01	0.00	0.01
Correction factor	2.33	2.68	4.01

N is the number of seal-biweeks that the correction factors were based on.

### Stock size estimates

We produced stock size estimates for the four regions of the study area, and the Northern Puget Sound and San Juans/Strait of Juan de Fuca regions combined provided a stock size estimate for the Washington Northern Inland Waters stock (Tables 3–5). The Southern Puget Sound stock was estimated to contain between 1,627 and 2,189 seals in different seasons, with an average seasonal estimate of 1,976 seals (CV = 20.53%). The Hood Canal stock was estimated to seasonally contain between 1,342 and 1,385 seals, with an average seasonal estimate of 1,368 seals (CV = 16.75%). For the Washington Northern Inland Waters stock, we could only estimate total stock size in spring, which is the only season that the waters of the San Juan Islands and Strait of Juan de Fuca were surveyed. We estimated that stock to contain 7,513 seals (CV = 11.50%).

**Table 3:** Density and abundance estimates for inland Washington harbor seals, Southern Puget Sound.

		Uncorrected Estimates				Corrected Estimates			
Region	Season	Density	Abundance	95%CI	CV	Density	Abundance	95% CI	%CV
S Puget Sound	Winter	0.62	280	144–544	21.6	nd	nd	nd	nd
	Spring	1.53	698	564–865	8.9	3.57	1627	1314–2015	8.97
	Summer	1.79	817	336–1990	35.9	4.80	2189	899–5330	35.9
	Autumn	1.16	526	357–777	16.3	4.64	2113	1429–3124	16.8
	Year-round	1.27	580	350–1044	20.7	4.34	1976	944–3490	20.5

**Table 4:** Density and abundance estimates for inland Washington harbor seals, Hood Canal.

		Uncorrected Estimates				Corrected Estimates			
Region	Season	Density	Abundance	95%CI	%CV	Density	Abundance	95% CI	%CV
Hood Canal	Winter	0.78	307	219–428	11.6	nd	nd	nd	nd
	Spring	1.51	590	445–782	11.59	3.52	1376	1038–1824	11.64
	Summer	1.28	501	316–796	18.53	3.43	1342	845–2132	18.55
	Autumn	0.88	345	219–544	19.68	3.54	1385	876–2187	20.05
	Year-round	1.11	436	300–638	15.35	3.50	1368	920–2048	16.75

**Table 5:** Density and abundance estimates for inland Washington harbor seals, Northern Inland Waters.

		Uncorrected Estimates				Corrected Estimates			
Region	Season	Density	Abundance	95%CI	CV	Density	Abundance	95% CI	%CV
N Puget Sound	Winter	0.42	756	575–995	13.23	nd	nd	nd	nd
	Spring	0.65	1156	921–1452	11.19	1.51	2694	2143–3386	11.24
	Summer	0.47	832	621–1114	14.31	1.24	2228	1663–2984	14.37
	Autumn	0.42	755	618–924	10.04	1.69	3032	2447–3757	10.75
	Year-round	0.49	875	684–1121	12.19	1.48	2651	2084–3376	12.12
San Juans/St. Juan de Fuca	Spring	0.44	2068	1512–2827	11.71	1.03	4819	3527–6584	11.76
WA N Inland Waters	Spring	0.47	3224	2433–4279	11.95	1.27	7513	5670–9970	11.5

## Discussion

### Potential biases of the estimates

There are several factors that can influence the accuracy of line-transect estimates of abundance [30]. These are generally related to the issues of ensuring representative survey coverage, incorporating seals that are missed on the surveys due to various factors, and the accuracy and precision of the correction factors. They can be addressed by taking steps to ensure that basic assumptions of distance-sampling theory are satisfied, or if they are not, to make certain that appropriate corrections are made. Each is discussed below.

With regard to obtaining representative survey coverage, transect lines were placed within the study area to sample the region in a representative fashion. Assumptions related to the accuracy of collected data were addressed by using clinometers to estimate sighting distances, thereby dramatically reducing opportunities for human errors related to rounding distance and angles to convenient values, etc.

**Fig 4:**
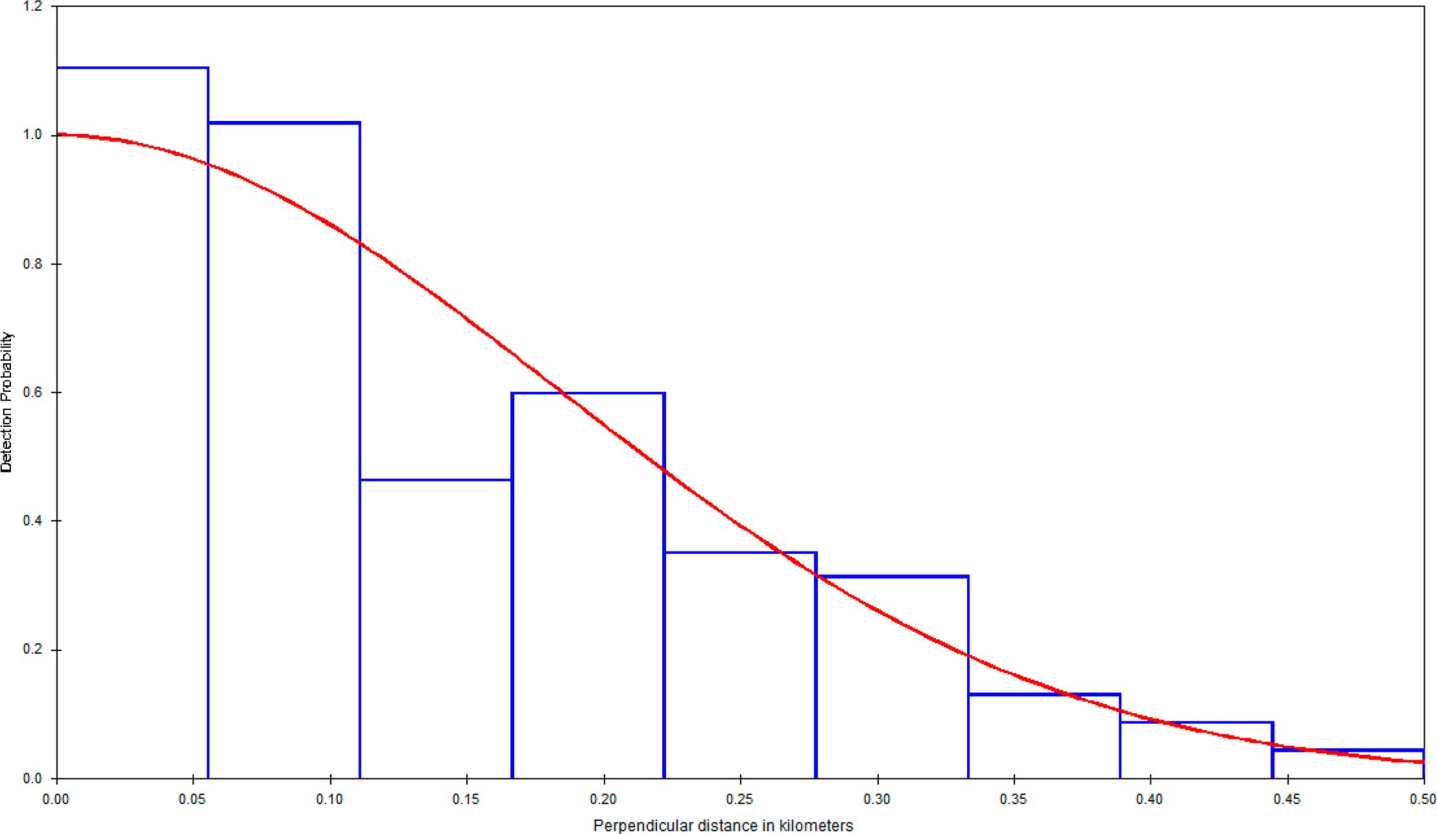
Perpendicular sighting distance histogram and fitted detection function (using a Half-Normal model with cosine adjustment), Southern Puget Sound.

**Fig 5:**
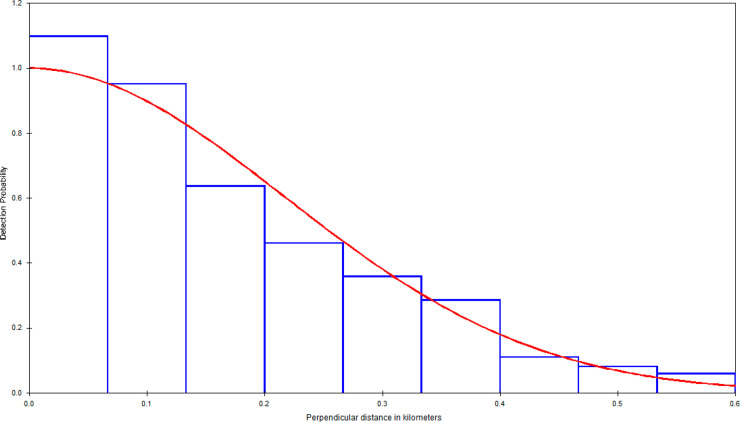
Perpendicular sighting distance histogram and fitted detection function (using a Half-Normal model with cosine adjustment), Hood Canal.

**Fig 6:**
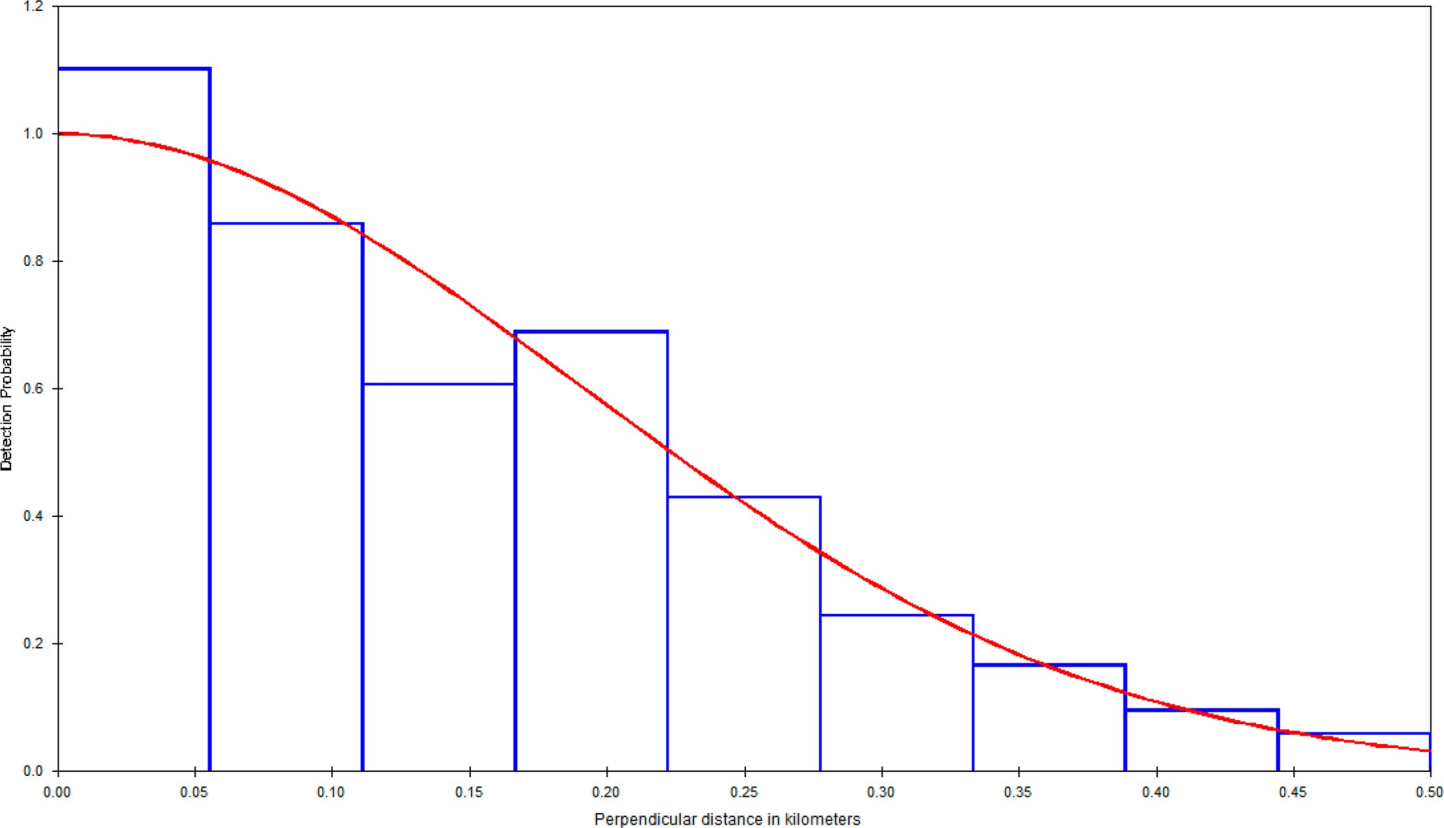
Perpendicular sighting distance histogram and fitted detection function (using a Half-Normal model with cosine adjustment), Northern Inland Waters.

A potential bias in our estimates comes from various factors that would result in seals not being detected during surveys. We feel that our uncorrected estimates should not suffer from any substantial upward or downward bias, since these simply refer to the number of seals that were available to be detected during our surveys. The use of a belly port window on the aircraft used in these surveys helped to ensure that target animals on and near the trackline were not missed to any significant degree. The PSD histograms (Figs 4–6) do not show any evidence of missed trackline detections, such as was the case in previous surveys in southern California, which used aircraft with no belly ports [51]. It should be noted that our trackline detection probability correction accounts for availability bias, but not for perception bias, which we do not expect to large.

A more significant potential bias is involved in the corrected estimates of stock size that we produced. These estimates must correct for two sources of missed seals. First, the proportion of seals that were on a dive as the aircraft passed overhead and would not be detected. Second, the proportion of seals that were hauled out on land during the surveys would also not be detected, since we did not conduct haul-out counts. Correcting for these two factors was a major focus of this study, and we consulted with a number of biologists in the region with extensive experience working with harbor seals. Because of non-continuous recording of wet-dry sensors on tags, we had to assume haul out probabilities were independent of dive probabilities. As tag technology is improved, continuous wet–dry sensors may help to generate more precise and less biased estimates of availability.

An additional factor to consider that may have influenced our stock size estimates is that our sample size for dive times was limited to only 15 tagged seals that may represent a non-random sample of seals with respect to behavior and demographics. With 10 males and 5 females, all adults, this sample may have somewhat of a sex skew toward male behavior, and may not fully reflect behavior of juveniles/pups. Similarly, because the tags were deployed to monitor behavior during overlap with outmigrating steelhead, this tagging effort is designed to represent behavior in spring and summer months, and may not be representative of behavior in other seasons.

Ultimately, we were able to correct most, but not all of our estimates, and this was due to the limited availability of data from other sources that were adequate to develop appropriate correction factors. Since line transect surveys are rarely used to estimate pinniped in-water abundance [39–42], we had no previous standard, and needed to develop a different approach. Fortunately, the availability of data from tagging studies on the same seal stocks that we were studying allowed us to develop a model that accounted for both sources of undetected seals.

Traditional pinniped haul-out counts are conducted at specific tidal states and as such require the relevant in-water correction factors corresponding to those same tidal states. Our line transect surveys were very different as they were not conducted according to any specific tidal state, were independent of tidal state, and therefore did not require any tidal state corrections. Our initial modeling included tidal heights and times to high tide (similar to London et al. [12]) as predictors. When the binomial GLMM predicting haul-out from the paper (with hour as fixed effects) was applied to these data, the AIC value (AIC = 29765.97) did not indicate any improvement. If instead of hour, time to high tide was used, the AIC became worse (29939.3) and the coefficient on the time to high tide was close to 0 and not significant (-0.0011758, SE = 0.0009015). The AIC and similar measures of predictive modeling accuracy are also worse when the time to high tide was used as a predictor.

It should be noted that the geographical area and seasonal focus are slightly different between the two studies, and this may have affected our results. In Hood Canal, where the London et al. [12] study was conducted, seals haul out at high tide, which is different from the timing in other parts of our study area. However, statistical modeling between the approaches was very similar (and the treatment of our covariates was largely motivated by London et al. [12]), so we do not expect this to be a significant source of discrepancy.

We feel that the corrected estimates produced in this paper are useful estimates of the total abundance for these stocks. However, the tagging studies had other goals and were not designed specifically to provide the kind of data that we were interested in. For example, factors such as seasonal variation (e.g., no data available for winter, and very little for autumn seasons) and age/sex class representation (e.g., tagging studies only cover larger seals, meaning that pups and juveniles will be unrepresented) are sources of uncertainty. In the inland waters of Washington State, the proportion of seals ashore at midday is known to vary dramatically throughout the year, from below 10% during the early summer pre-pupping season to as much as 60–70% during the pupping and molting seasons in late summer and autumn [4, 12, 52]. A refined understanding of how these proportions for all age/sex classes of seals change throughout the day and year, and how this information can be most effectively incorporated into relevant correction factors is desirable. Suboptimal weather can certainly affect our estimates, but our approach of using only data collected under good sighting condition (Beau 0–2 and periods without significant glare) minimized this. We feel that we have made significant progress, but we urge further work, in both field studies and theoretical development, to address these issues, as well as concerns about potential bias in methods that are not close to being instantaneous [53].

### Comparison to previous estimates

In the early part of the twentieth century, the State of Washington conducted a predator control program, largely to protect commercial and sport fisheries, and fish hatcheries [3]. This program involved putting a bounty on the heads of pinnipeds. Harbor seal numbers in the state were dramatically reduced by these measures [4]. However, the bounty system was ended in 1960, and in 1972 the MMPA gave widespread protection to all marine mammal species. Harbor seal numbers rose as a result [4, 5].

In the early 1940s, Scheffer and Slipp [3] estimated that there were between 5,000 and 10,000 harbor seals in Washington State. In 1970–1972 (before enactment of the MMPA), haul-out counts of harbor seals in inland Washington waters (uncorrected for animals in the water) were only about 1,400 animals [4]. However, by 1977–1978, the numbers were thought to be over 2,300 seals [51]. By the early 1990s, counts (now corrected with a factor to account for seals in the water) ranged from about 12,000 to 16,000 seals [54]. The most recent complete estimates of the Washington inland waters stocks, based on 1999 counts, and corrected for in-water seals, gave a total of 13,692 seals (with 1,088 in Hood Canal, 1,568 in Southern Puget Sound, and 11,036 in Washington Northern Inland Waters [5]). There are more recent estimates for specific subareas, such as Hood Canal and the San Juan Islands (see Table 6), and these have generally supported the idea that the three inland Washington stocks had reached some level approaching stability [4, 5].

**Table 6:** Estimates of abundance and stock size for harbor seals in the inland waters of Washington.

Years	Area	Type	Estimate (CI or %CV)	Stock Est?	Reference
1970–1972	Total WA Inland Waters	Uncorr.Count	1386 (1033–1807)		[4]
1977–1978	Hood Canal	Uncorr.Count	732 (n/a)	X	[52]
1977–1978	Southern Puget Sound	Uncorr.Count	337 (n/a)	X	[52]
1977–1978	Northern Puget Sound	Uncorr.Count	1237 (n/a)		[52]
1991	Total WA Inland Waters	Corr. Count	12,883 (11,135–14,906)		[54]
1992	Total WA Inland Waters	Corr. Count	13,516 (11,743–15,555)		[54]
1993	Total WA Inland Waters	Corr. Count	16,253 (14,220–18,577)		[54]
1999	Hood Canal	Corr. Count	1088 (CV = 0.15)	X	[5]
1999	Southern Puget Sound	Corr. Count	1568 (CV = 0.15)	X	[5]
1999	WA Northern Inland Waters	Corr. Count	11,036 (CV = 0.15)	X	[5]
1999	Total WA Inland Waters	Corr. Count	13,692 (n/a)		[5]
2002	Hood Canal	Corr. Count	1068 (n/a)	X	[65]
2013–2016	Hood Canal	LT in-water only	2009 (1750–2308)	X	[22]
2013–2016	Hood Canal	LT corrected	1368 (920–2048)	X	This study
2013–2016	Southern Puget Sound	LT corrected	1976 (944–3490)	X	This study
2013–2016	WA Northern Inland Waters	LT corrected	7513 (5670–9970)	X	This study
2013–2016	Total WA Inland Waters	LT corrected	10,857 (7534–15,508)		This study
2019	San Juan Islands	Corr. Count	5202 (n/a)		[55]

Estimates are from either aerial survey counts or line-transect (LT) surveys, and either provided uncorrected haul-out counts or counts corrected for animals in the water. CI is 95% confidence interval, CV is coefficient of variation.

Although the use of different survey and analysis methods makes direct comparison of various estimates challenging, it is still instructive to examine our stock size estimates in relation to previous estimates that also attempted to derive complete estimates of those stocks. Our estimates from line-transect aerial surveys of in-water seals, and corrected for both missed seals that were diving and those that were onshore during our surveys (i.e., 1,368 in Hood Canal, 1,976 in Southern Puget Sound, and 7,513 in Washington Northern Inland Waters), suggest that the overall Washington inland waters harbor seal numbers may have declined somewhat since 1999 (Table 6). If this is true, it appears to be mostly related to a large drop in the numbers of seals in the Washington Northern Inland Waters stock. Our estimates for the Hood Canal and Southern Puget Sound stocks, on the other hand, suggest that those stocks may have increased somewhat since 1999 and the early 2000s (see Table 6).

### Usefulness of our methods

Although several methods have been used to estimate the abundance and population size of pinnipeds, in most cases some modification of a colony count or haul-out count is applied [56, 57]. This often involves direct counts of the number of seals onshore at a particular time (usually during the breeding season, when the highest proportion is onshore), with other techniques then used to correct for the number of animals that were missed or were in the water. Such methods have become standard practice for size estimation of most pinniped stocks.

As discussed above, distance-sampling methods (e.g., line-transect and strip-transect sampling) have rarely been used to estimate abundance of pinnipeds, though there appears to be a trend toward increasing use of these methods. While their use for cetaceans is relatively straightforward, pinnipeds add the additional complexity that there is never a time a when all of the animals are onshore, or all in the water, and the proportion of these two parameters can vary dramatically with time of day, season, tidal condition, etc. [12, 58]. While our correction factors are specific to Washington inland waters harbor seals, a similar approach can be used for many other areas where there have been similar seal tagging studies. For many situations, it may require directed studies to develop the appropriate correction factors.

In recent decades, line-transect methods have been used to survey for seals on solid substrates, such as land or sea ice. These studies therefore do not use line-transect methods to estimate in-water densities [37, 38, 40, 42, 48, 59]. In other cases, uncorrected estimates of in-water only density have been made using line-transect methods [39, 41]. However, neither of these types of studies can provide total estimates of abundance or stock size.

Our methods presented here have utility for many species of pinnipeds and exhibit one notable advantage, especially when there is a desire to obtain information on multiple species with one survey. That is multi-species surveys can be conducted vs. just targeting a single species of pinniped. In fact, in our surveys in Washington State, we collected data on all species of marine mammals observed, and estimates of line-transect abundance were also made for harbor porpoises [23], and sea lions (California sea lions and Steller *Eumetopias jubatus* sea lions combined; 21]. A minor disadvantage is the need to develop accurate and precise correction factors to account not only for animals that were diving, but also for those that were on shore during the surveys. Williams and Thomas [60] and Best et al. [44] used a novel approach, which involved collecting data to estimate numbers of animals in the water and numbers onshore at the same time, both using line-transect methods. Where feasible, this method shows much promise. Even more promising would be a combined survey with two aircraft, one of which conducted line-transect surveys to estimate in-water abundance, while the other conducted simultaneous colony counts in the same area. Lowry and Forney [46] have recently demonstrated the utility of the latter approach for California sea lions.

### Management implications

If our estimates presented here are indeed accurate, then comparison with previous estimates from 1999 [5] would lead one to conclude that harbor seal stocks in both Hood Canal and Southern Puget Sound have increased somewhat, while the Washington Northern Inland Waters stock has decreased. Both the 1999 surveys and ours were analyzed with the goal of obtaining total numerical size for these seal stocks. Comparing abundance estimates generated by the two different methods should only be done with the recognition that each may include unknown bias. If we assume both estimates are accurate, it is worth discussing why stock sizes may have changed from 1999 to the time of this study. Although population dynamics of harbor seals are complex, involving complicated ecological interactions with their predators and prey, their habitat, and numerous anthropogenic factors, predation pressure may be key here.

Transient or Bigg’s killer whales (*Orcinus orca*) are common in parts of the Salish Sea, and they feed almost exclusively on marine mammals [61]. Harbor seals are a major prey item of killer whales in this area [61], and since at least 2004, Bigg’s killer whales have been spending increasing amounts of time in the Salish Sea [62, 63]. In fact, the scale of this increase in numbers and occupancy has steepened in recent years, with 2017 reaching a peak of over 200 occurrences [64].

Salish Sea Bigg’s killer whales are distributed mainly in open-ended waterways such as the region around the San Juan Islands and to a lesser extent the Strait of Juan de Fuca; their visits to Hood Canal and Southern Puget Sound (both dead-end channels) are relatively infrequent and generally involve smaller groups of killer whales [64, 65]. Since mammal-eating killer whales are spending significantly more time in the northern inland waters of Washington, it is likely that harbor seal predation has also increased there. Shields et al. [63] recently estimated that Bigg’s killer whales in the Salish Sea consumed well over 1,090 harbor seals in 2017. If these were mainly taken from the Washington Northern Inland Waters stock, then this could certainly have an impact on that stock, with little to no effect on the stocks in Hood Canal and Southern Puget Sound, where the killer whales rarely feed.

A wide range of marine birds and mammals prey on salmon in the marine environment. In the inland waters of the Salish Sea, predation by harbor seals has become increasingly evident through new techniques to identify prey in scat [16, 66] and other recently developed techniques to detect predation events through different tag technologies [67, 68]. Declines in some anadromous salmonids and marine survival rates have coincided with increased harbor seal abundance over the past 40–50 years, although these associations and estimates based on bioenergetics and fisheries data do not provide a mechanistic link [17, 69]. Harbor seals are estimated to consume more Chinook salmon than other marine mammal predators (e.g., Steller and California sea lions, and killer whales), largely because they consume more numerous smolts migrating to the Pacific Ocean, and resident salmon that remain in Puget Sound year-round [15]. Harbor seal abundance is just one of many factors that may influence predation on juvenile and adult anadromous salmonids and their impacts on ESA-listed populations. The abundance and distributions of preferred prey items such as hake, herring, cod, and other species [14] with highly variable recruitment, abundance, and spatial distributions in the Salish Sea (e.g., anchovies) [70] may alter predation pressure by harbor seals on anadromous salmonids. With due consideration of the numerous factors that influence predation by harbor seals on juvenile and adult salmonids, the present study indicates that harbor seal abundance in some areas may have increased somewhat from the Jeffries et al. [4] estimate and suggests continued, or even increased, predation impacts on Puget Sound anadromous salmonid populations.

## Conclusions

Our estimates of abundance for harbor seals in the inland waters of Washington have value for managers, who are currently limited by the absence of up-to-date abundance estimates for these stocks. Since our estimates are from the 2013–16 period, they provide a much more recent stock assessment for the three stocks recognized by NMFS than the previously-available estimates, which are now over 21 years old [5]. They will also be useful in evaluating the impacts of predation by seals on the various fish stocks of concern.

Finally, we are hopeful that the methods used in this study will be found to have value as an alternative approach to estimating pinniped abundance. Our methods take a “reverse” approach from the traditional methods, which use aerial counts of seal haul-outs, correcting for seals in the water. Ours also have the advantage of providing density estimates, which are not available from traditional haul-out counts. They represent a different approach to providing estimates of the entire population/stock size of amphibious marine mammal species. We feel that they show promise for widespread use, especially for situations in which multiple marine mammal species are surveyed in the same project.

## References

[1] Gaydos JKG, Pearson S. Birds and mammals that depend on the Salish Sea: A compilation. Northw. Nat. 2011; 92:79–94.

[2] Zier J, Gaydos JK. Harbor seal species profile: Puget Sound Institute at the University of Washington, Tacoma; 2014. p. 1–55]. Available from: https://www.eopugetsound.org/.

[3] Scheffer VB, Slipp JW. The harbor seal in Washington State. Am Midl Nat. 1944; 32:373–416.

[4] Jeffries S, Huber H, Calambokidis J, Laake J. Trends and status of harbor seals in Washington State: 1978–1999. J Wildl Manage. 2003; 67:208–19.

[5] Carretta JV, Forney KA, Oleson EM, Weller DW, Lang AR, Baker J, et al. U.S. Pacific marine mammal stock assessments: 2019. NOAA Technical Memorandum NMFS-SWFSC. 2020; 629:380 p.

[6] Barlow J, Swartz SL, Eagle TC, Wade PR. U.S. marine mammal stock assessments: guidelines for preparation, background, and a summary of the 1995 assessments. NOAA Technical Memorandum NMFS-OPR. 1995; 6:73 p.

[7] Jeffries SJ, Gearin PJ, Huber HR, Saul DL, Pruett DA. Atlas of Sea and Sea Lion Haulout Sites in Washington: Washington Department of Fish and Wildlife; 2000. 139 p.

[8] Suryan RM, Harvey JT. Tracking harbor seals (*Phoca vitulina richardsi*) to determine dive behavior, foraging activity, and haul-out site use. Mar Mamm Sci. 1998; 14(2):361–72.

[9] Hardee SE. Movements and home ranges of harbor seals (*Phoca vitulina*) in the inland waters of the Pacific Northwest: MS thesis, Western Washington University, Bellingham, WA USA; 2008.

[10] Department of Fisheries and Oceans. Population Assessment—Pacific Harbour Seal (*Phoca vitulina richardsi*). Canadian Science Advisory Secretariat Science Advisory Report. 2010; 2009/011, 10 p.

[11] Acevedo G. A, Cendejas-Zarelli S. Nocturnal haul-out patterns of harbor seals (*Phoca vitulina*) related to airborne noise levels in Bellingham, Washington, USA. Aquat Mamm. 2011; 37(2):167–74.

[12] London JM, Ver Hoef JM, Jeffries SJ, Lance MM, Boveng PL. Haul-out behavior of harbor seals (*Phoca vitulina*) in Hood Canal, Washington. Plos One. 2012; 7(6(e38180)):9 p. doi: 10.1371/journal.pone.0038180 22723851 PMC3377645

[13] Lance MM, Chang WY, Jeffries SJ, Pearson SF, Acevedo-Gutierrez A. Harbor seal diet in northern puget sound: Implications for the recovery of depressed fish stocks. Mar.Ecol. Prog. Ser. 2012; 464: 257–271. doi: 10.3354/meps09880

[14] Thomas AC, Lance MM, Jeffries SJ, Miner BG, Acevedo-Gutiérrez A. Harbor seal foraging response to a seasonal resource pulse, spawning Pacific herring. Mar Ecol Prog Ser. 2011; 441:225–39. doi: 10.3354/meps09370

[15] Chasco B, Kaplan IC, Thomas A, Acevedo-Gutierrez A, Noren D, Ford MJ, et al. Estimates of chinook salmon consumption in Washington state inland waters by four marine mammal predators from 1970 to 2015. Can. J. Fish. Aquat. Sci. 2017; 74:1173–1194. doi: 10.1139/cjfas-2016-0203

[16] Thomas AC, Nelson BW, Lance MM, Deagle BE, Trites AW. Harbour seals target juvenile salmon of conservation concern. Can J Fish Aquat Sci. 2017; 74(6):907–21. doi: 10.1139/cjfas-2015-0558

[17] Nelson BW, Walters CJ, Trites AW, McAllister MK. 2019. Wild chinook salmon productivity is negatively related to seal density and not related to hatchery releases in the pacific northwest. Can. J. Fish. Aquat. Sci. 2019; 76:447–462. doi: 10.1139/cjfas-2017-0481

[18] Gearin P, Pfeifer B, Jeffries S. Control of California sea lion predation of winter-run steelhead at the Hiram M. Chittenden Locks, Seattle, December 1985-April 1986. Washington Department of Game and Fisheries Management Report. 1986; 86–20:108 p.

[19] Marshall KN, Stier AC, Samhouri JF, Kelly RP, Ward EJ. Conservation challenges of predator recovery. Conser. Lett. 2016; 9:70–78. doi: 10.1111/conl.12186

[20] Daniel RG, Jemison LA, Pendleton GW, Crowley SM. Molting phenology of harbor seals on Tugidak Island, Alaska. Mar Mamm Sci. 2003;19(1):128–40.

[21] Smultea, MA, Lomac-MacNair K, Campbell G, Courbis SS, Jefferson TA. Aerial Surveys of Marine Mammals Conducted in the Inland Puget Sound Waters of Washington, Summer 2013–Winter 2016. Final Report. Prepared for Commander, U.S. Pacific Fleet and Naval Sea Systems Command. Submitted to Naval Facilities Engineering Command Pacific, Pearl Harbor, Hawaii under Contract No. N62470-15-D-8006, Task Order KB05 issued to HDR, San Diego, CA, 76+ p. 2017.

[22] Ampela K, Jefferson TA, Smultea MA. Estimation of in-water density and abundance of harbor seals. J. Wildl. Manage. 2021; 2021:1–7. doi: 10.1002/jwmg22019

[23] Jefferson TA, Smultea MA, Courbis SS, Campbell GS. Harbor porpoise (*Phocoena phocoena*) recovery in the inland waters of Washington: Estimates of density and abundance from aerial surveys, 2013–2015. Canadian Journal of Zoology. 2016; 94:505–15.

[24] Temte JL. Photoperiod and the timing of pupping in the Pacific harbor seal (*Phoca vitulina richardsi*) with notes on reproduction in northern fur seals and Dall porpoises. MS Thesis, Oregon State University, Corvallis, OR USA; Xiii + 147pp 1986.

[25] Jeffries SJ. Occurrence and distribution patterns of marine mammals in the Columbia River and adjacent coastal waters of northern Oregon and Washington. Pages 15–50 in Marine Mammals and Their Interactions with Fisheries of the Columbia River and Adjacent Waters 1980–1982. NWAFC Processed Report 1985; 85–04, 93 p.

[26] Brown RF. Assessment of pinniped populations in Oregon: April 1984 to April 1985. NWAFC Processed Report 1988; 88–05, 44 p.

[27] Calambokidis J, Speich SM, Peard J, Steiger GH, Cubbage JC. Biology of Puget Sound marine mammals and marine birds: population health and evidence of pollution effects. NOAA Technical Memorandum NOS-OMA. 1985; 18:159 p.

[28] Huber HR, Jeffries SJ, Lambourn DM, Dickerson BR. Population substructure of harbor seals (*Phoca vitulina richardsi*) in Washington State using mtDNA. Canadian Journal of Zoology 2010; 88(3):280–288.

[29] Huber HR, Dickerson BR, Jeffries SJ, Lambourn DM. Genetic analysis of Washington State harbor seals (*Phoca vitulina richardii*) using microsatellites. Canadian Journal of Zoology 2012; 90(12):1361–1369.

[30] Buckland ST, Anderson DR, Burnham KP, Laake JL, Borchers DL, Thomas L. Introduction to Distance Sampling: Estimating Abundance of Biological Populations: Oxford University Press; 2001. 432 p.

[31] Buckland ST, Anderson DR, Burnham KP, Laake JL, Borchers DL, Thomas L. Advanced Distance Sampling: Oxford University Press; 2004. 416 p.

[32] Marques FFC, Buckland ST. Covariate models for the detection function. In: Buckland ST, Anderson DR, Burnham KP, Laake JL, Borchers DL, Thomas L, editors. Advanced Distance Sampling: Oxford University Press; 2004. p. 31–47.

[33] Marques FFC, Buckland ST. Incorporating covariates into standard line transect analyses. Biometrics. 2003; 59:924–35. doi: 10.1111/j.0006-341x.2003.00107.x 14969471

[34] Thomas L, Buckland ST, Rexstad EA, Laake JL, Strindberg S, Hedley SL, et al. Distance software: design and analysis of distance sampling surveys for estimating population size. J Appl Ecol. 2010; 47:5–14. doi: 10.1111/j.1365-2664.2009.01737.x 20383262 PMC2847204

[35] Moore ME, Berejikian BA, Greene CM, Munsch S. Environmental fluctuation and shifting predation pressure contribute to substantial variation in early marine survival of steelhead. Marine Ecology Progress Series 2021; 662:139–156.

[36] Lunn NJ, Stirling I, Nowicki SN. Distribution and abundance of ringed (*Phoca hispida*) and bearded seals (*Erignathus barbatus*) in western Hudson Bay. Can J Fish Aquat Sci. 1997; 54:914–21.

[37] Mizuno AW, Wada A, Ishinazaki T, Hattori K, Watanabe Y, Ohstaishi N. Distribution and abundance of spotted seals *Phoca largha* and ribbon seals *Phoca fasciata* in the southern Sea of Okhotsk. Ecological Research 2002; 17(1):79–96.

[38] Chambellant M, Ferguson SH. Comparison of strip- and line-transect sampling to estimate density and abundance of ringed seals (*Phoca hispida*) in western Hudson Bay, 2007 and 2008. Canadian Science Advisory Secretariat Research Document 2009; 2009/002 Iii + 19p.

[39] Herr H, Scheidat M, Lehnert K, Siebert U. Seals at sea: modelling seal distribution in the German bight based on aerial survey data. Marine Biology (Berlin) 2009; 156(5):811–820 2009.

[40] Bengston JL, Laake JL, Boveng PL, Cameron MF, Hanson MB, Stewart BS. Distribution, density, and abundance of pack-ice seals in the Amundsen and Ross Seas, Antarctica. Deep Sea Research Part Ii: Topical Studies In Oceanography 2011; 58(9–10):1261–1276 2011.

[41] Buckland ST, Cattanach KL, Hobbs RC. Abundance estimates of Pacific white-sided dolphin, northern right whale dolphin, Dall’s porpoise and northern fur seal in the North Pacific, 1987–1990. International North Pacific Fisheries Commission Bulletin. 1993; 53(3):387–407.

[42] Gelatt TS, Siniff DB. Line transect survey of crabeater seals in the Amundsen-Bellingshausen Seas, 1994. (*Lobodon carcinophagus*). Wildlife Society Bulletin 1999; 27(2):330–336.

[43] Aerts LAM, McFarland AE, Watts BH, Lomac-MacNair KS, Seiser PE, Wisdom SS, et al. Marine mammal distribution and abundance in an offshore sub-region of the northeastern Chukchi Sea during the open-water season. Cont Shelf Res. 2013; 67:116–26.

[44] Best BD, Fox CH, Williams R, Halpin PN, Paquet PC. Updated marine mammal distribution and abundance estimates in British Columbia. J Cetacean Res Manage. 2015; 15:9–26.

[45] Carretta JV, Lowry MS, Stinchcomb CE, Lynne MS, Cosgrove RE. Distribution and abundance of marine mammals at San Clemente Island and surrounding offshore waters: results from aerial and ground surveys in 1998 and 1999. Southwest Fisheries Science Center Administrative Report. 2000; LJ-00-02:43 p.

[46] Lowry MS, Forney KA. Abundance and distribution of California sea lions (*Zalophus californianus*) in central and northern California during 1998 and summer 1999. Fish Bull. 2005; 103:331–43.

[47] Southwell CJ, de la Mare B, Borchers D, Burt L. Shipboard line transect surveys of crabeater seal abundance in the pack-ice off east Antarctica: Evaluation of assumptions. Mar Mamm Sci. 2004; 20(3):602–20.

[48] Ver Hoef J, Cameron MF, Boveng PL, London JM, Moreland EE. A spatial hierarchical model for abundance of three ice-associated seal species in the eastern Bering Sea. Statistical Methodology 2013; 17:46–66 2013.

[49] Wilson K, Lance M, Jeffries SJ, Acevedo-Gutierez A. Fine-scale variability in harbor seal foraging behavior. PLos ONE. 2014; 9 (e92838):1–13. 10.1371/journal.pone.0092838 PMC3981695 24717815

[50] Brooks ME, Kristensen K, van Benthem KJ, Magnusson A, Berg CW, Nielsen A, et al. glmmTMB balances speed and flexibility among packages for zero-inflated generalized linear mixed modeling. R Journal. 2017; 9:378–400.

[51] Jefferson TA, Smultea MA, Bacon CE. Southern California Bight marine mammal density and abundance from aerial surveys, 2008–2013. Journal of Marine Animals and Their Ecology. 2015 (2014); 7:14–30.

[52] Calambokidis JA, Everitt RD, Cubbage JC, Carter SD. Harbor seal census for the inland waters of Washington, 1977–1978. (*Phoca vitulina*). Murrelet 1979; 60(3):110–112.

[53] Borchers DL, Zucchini W, Heide-Jorgensen MP, Canadas A, Langrock R. Using hidden Markov models to deal with availability bias on line transect surveys. Biometrics. 2013; 69(3):703–13. Epub 2013/07/16. doi: 10.1111/biom.12049 . 23848543

[54] Huber HR, Jeffries SJ, Brown RF, DeLong RL, VanBlaricom G. Correcting aerial survey counts of harbor seals (*Phoca vitulina richardsi*) in Washington and Oregon. Mar Mamm Sci. 2001; 17(2):276–93.

[55] Ashley EA, Olson JK, Adler TE, Raverty S, Anderson EM, Jeffries SJ, et al. Causes of mortality in a harbor seal (*Phoca vitulina*) population at equilibrium. Frontiers in Marine Science. 2020; 7. doi: 10.3389/fmars

[56] Hammond PS. Estimating the abundance of marine mammals: a North Atlantic perspective. In: Blix AS, WallOe L, Ultang O, editors. Whales, Seals, Fish and Man: Elsevier Science; 1995; p. 3–12.

[57] Buckland ST, York AE. Abundance estimation. In Encyclopedia of Marine Mammals, 3^rd^ ed. (Würsig B., Thewissen J. G. M., Kovacs K. M., eds.), 2018; p. 1–6. Elsevier/Academic Press.

[58] Patterson J, Acevedo-Gutierrez A. Tidal influence on the haul-out behavior of harbor seals (*Phoca vitulina*) at a site available at all tide levels. Northwest Nat. 2008; 89:17–23.

[59] Southwell C, Borchers D, Paxton CGM, Burt L, De La Mare WK. Estimation of detection probability in aerial surveys of Antarctic pack-ice seals. Journal of Agricultural, Biological And Environmental Statistics 2007; 12(1):41–54.

[60] Williams R, Thomas L. Distribution and abundance of marine mammals in the coastal waters of British Columbia, Canada. J Cetacean Res Manage. 2007; 9:15–28.

[61] Ford JKB, Ellis GM, Barrett-Lennard LG, Morton AB, Palm RS, Balcomb KC III. Dietary specialization in two sympatric populations of killer whales (*Orcinus orca*) in coastal British Columbia and adjacent waters. Canadian Journal of Zoology. 1998; 76:1456–71.

[62] Houghton J, Baird RW, Emmons CK, Hanson MB. Changes in the Occurrence and Behavior of Mammal-eating Killer Whales in Southern British Columbia and Washington State, 1987–2010. Northwest Sci. 2015; 89(2):154–69.

[63] Shields MW, Hysong-Shimazu S, Shields JC, Woodruff J. Increased presence of mammal-eating killer whales in the Salish Sea with implications for predator-prey dynamics. 2018; PeerJ 6:e6062. doi: 10.7717/peerj.6062 30564522 PMC6284519

[64] Shields MW, Viers S. Status and trends for West Coast transient (Bigg’s) killer whales in the Salish Sea. Encyclopedia of Puget Sound at the University of Washington Puget Sound Institute. 2019; Available at https://www.eopugetsound.org/articles/biggs-killer-whales, accessed 29 June 2020.

[65] London JM. Harbor Seals in Hood Canal: Predators and Prey: PhD thesis, University of Washington, Seattle, WA USA; 2006.

[66] Deagle BE, Thomas AC, McInnes JC, Clarke LJ, Vesterinen EJ, Clare EL, et al. Counting with DNA in metabarcoding studies: How should we convert sequence reads to dietary data? Mol. Ecol. 2019; 28:391–406. doi: 10.1111/mec.14734 29858539 PMC6905394

[67] Berejikian BA, Moore ME, Jeffries SJ. Predator-prey interactions between harbor seals and migrating steelhead trout smolts revealed by acoustic telemetry. Mar Ecol Prog Ser. 2016; 543:21–35. doi: 10.3354/meps11579

[68] Allegue H, Thoma AC, Liu Y, Trites AW. Harbour seals responded differently to pulses of out-migrating coho and Chinook smolts. Mar. Ecol. Prog. Ser. 2020; 647:211–227.

[69] Sobocinski KL., Kendall NW, Greene CM, Schmidt MW. Ecosystem indicators of marine survival in Puget Sound steelhead trout. Progress in Oceanography. In press.

[70] Duguid WDP, Bold JL, Chalifour L, Greene CM, Galbraith MJ, Hay D, et al. Historical fluctuations and recent observations of northern anchovy *Engraulis mordax* in the Salish Sea. Deep-Sea Research Part II-Topical Studies in Oceanography 2019; 159: 22–41. doi: 10.1016/j.dsr2.2018.05.018

